# Long-term air pollution levels modify the relationships between short-term exposure to meteorological factors, air pollution and the incidence of hand, foot and mouth disease in children: a DLNM-based multicity time series study in Sichuan Province, China

**DOI:** 10.1186/s12889-022-13890-7

**Published:** 2022-08-04

**Authors:** Caiying Luo, Jian Qian, Yaqiong Liu, Qiang Lv, Yue Ma, Fei Yin

**Affiliations:** 1 West China School of Public Health and West China Fourth Hospital, Sichuan University, Sichuan Chengdu, China; 2 Sichuan Center for Disease Control and Prevention, Chengdu, Sichuan China

**Keywords:** Hand, foot, and mouth disease, Air pollution, Environmental factors-HFMD association, Modification effect, Multicity analysis

## Abstract

**Background:**

Epidemiological studies have investigated the short-term effects of meteorological factors and air pollution on the incidence of hand, foot, and mouth disease (HFMD). Several meteorological indicators, such as relative humidity and the diurnal temperature range (DTR), significantly modify the relationship between short-term exposure to temperature and HFMD incidence. However, it remains unclear whether (and how) long-term air pollution levels modify the short-term relationships of HFMD incidence with meteorological factors and air pollution.

**Methods:**

We obtained daily data on meteorological factors, air pollutants, and HFMD counts in children from 21 prefecture-level cities in Sichuan Province in Southwest China from 2015 to 2017. First, we constructed a distributed lag nonlinear model (DLNM) at each prefecture-level site to evaluate the short-term impacts of meteorological variables and air pollutants on HFMD incidence. Then, we assessed the pooled effects of the exposures and incorporated long-term city-specific air pollutant indicators as meta-predictors to examine their potential modification effects by performing multivariate meta-regression models.

**Results:**

We found that long-term SO_2_ and CO concentrations significantly modified the short-term relationships between climatic variables and HFMD incidence. Specifically, high concentrations of CO (*P* = 0.027) and SO_2_ (*P* = 0.039) reduced the risk of HFMD at low temperatures. The relationship between relative humidity and HFMD incidence was weakened at high SO_2_ concentrations (*P* = 0.024), especially when the relative humidity was below the median level. When the minimum relative humidity (32%) was compared to the median relative humidity (77%), the risk ratio (RR) was 0.77 (95% CI: 0.51–1.17) in the 90^th^ percentile of SO_2_ (19.6 μg/m^3^) and 0.41 (95% CI: 0.27–0.64) in the 10^th^ percentile of SO_2_ (10.6 μg/m^3^).

**Conclusion:**

Our results indicated that long-term SO_2_ and CO levels modified the short-term associations between HFMD incidence in children and meteorological variables. These findings may inform health authorities to optimize targeted public health policies including reducing ambient air pollution and reinforcing self-protective actions to weaken the adverse health impacts of environmental factors on HFMD incidence.

**Supplementary Information:**

The online version contains supplementary material available at 10.1186/s12889-022-13890-7.

## Background

Hand, foot, and mouth disease (HFMD), an infectious disease caused by several human enteroviruses, primarily affects the physical and mental health of children less than 5 years of age [1]. One of the notable routes of HFMD transmission is through respiratory secretions [2], as patients have a relatively strong ability to transmit enteroviruses to the environment and other vulnerable populations. Over the last few decades, HFMD has become a prominent public health problem in numerous countries in the Asia–Pacific region [3, 4]. The HFMD disease burden remains high, especially in China. The annual reported number of HFMD cases has reached 1.61 to 2.77 million [5]. To date, there are no specific antienterovirus agents administered for HFMD. Since 2016, three EV-A71 vaccines, which are effective in protecting against only EV-A71-associated HFMD infections [6], have been licensed in China [7]. However, the dominant serotypes have shifted to CV-A6 and CV-A10, which the existing vaccines provide no protection against [6]. The incidence of HFMD remained high even after implementing vaccination measures. In 2018, the incidence rate of HFMD was 169.41/100,000 population; this incidence rate was much higher than those of other notifiable infectious diseases, which ranged from < 0.00/100,000 to 92.31/100,000 [8].

To understand the transmission characteristics of HFMD, and thereby control the disease, many studies have investigated its risk factors. Meteorological factors, especially the ambient temperature and relative humidity, are the leading causes of negative health consequences [9–11]. Indeed, the heterogeneous findings among several multisite studies demonstrate that location-specific characteristics, such as climate conditions, play important roles in the meteorological factor-HFMD association [10, 12–14]. In addition, epidemiological studies have found a significant impact of air pollution on childhood respiratory diseases, such as asthma [15, 16]. To date, the short-term effects of air pollution on HFMD incidence have been explored by limited studies, and the findings from different regions are inconsistent. For instance, a study conducted in Ningbo, a coastal city in eastern China, did not find a significant relationship between short-term exposure to particulate matter 10 (PM_10_; with a diameter ≤ 10 microns) and HFMD incidence [17]. In contrast, another study reported an inverted V-shaped pattern described the relationship between PM_10_ levels and HFMD incidence in inland Chengdu, a typical basin city in southwestern China [18]. Similarly, Yu et al. found that short-term exposure to low O_3_ concentrations was related to an increased risk of HFMD in Guilin, China [19]. However, studies in coastal Shenzhen [20] and Ningbo [21] revealed generally M-shaped and inverted V-shaped curves, respectively. These inconsistent results might be attributed to the influence of location-specific factors, such as environmental variables. Moreover, studies that have explored the potentially nonlinear relationships between HFMD incidence and short-term exposure to air pollutants have only constructed single-city time-series regressions, limiting the evaluation and explanation of the heterogeneity. Therefore, a multicity analysis that is more appropriate for addressing heterogeneity should be performed to gain a comprehensive understanding of the relationship between air pollution and HFMD incidence.

With the increasing interest in the health effects of climate change, concerns have been raised regarding the joint effects of environmental factors, including climatic variables and air pollution, on health. Epidemiological evidence has suggested that the modification effects of meteorological and air pollution variables on short-term mortality effects is of great value to public health [22, 23]. Several studies have estimated the modification effects of long-term meteorological indicators on the associations of HFMD incidence with relative humidity and temperature [10, 13, 24]. However, the health impacts of air pollution on HFMD incidence have been quantified by only exposure-lag-disease associations [19, 21]. Alternatively, air pollutants are typically controlled for as confounders instead of modifiers [17, 18]. The potential for long-term air pollution levels to modify the relationships between HFMD incidence and environmental factors (including meteorological and air pollution variables) has been ignored in environmental epidemiological studies thus far. Hence, this study assessed the modification effects of air pollutants on the associations between short-term exposure to environmental factors and HFMD incidence, providing novel insights into the health impacts of air pollution.

Sichuan Province was chosen as the study area for three reasons. First, this province has a highly complex topography, with a distinct basin in the eastern region and a variety of environmental conditions across the prefecture-level cities. Second, the complex topography and unique climatic conditions have markedly reduced the self-cleansing of the atmosphere in the basin region. Due to the frequent and severe air pollution events that persist for long durations, the Sichuan Basin is one of the four most heavily polluted areas in China [25]. Finally, HFMD incidence in Sichuan Province is high and gradually increasing, which might facilitate the identification of factors that influence health risks and the joint effects of exposures.

This study conducted the first assessment of whether long-term air pollution levels modify the relationships of short-term exposure to climatic variables and air pollution with HFMD incidence using a multisite modeling framework in a typical area with a complex terrain, diverse climatic conditions, and severe air pollution. The first aim of our present study was to estimate the short-term health impacts of meteorological variables (i.e., temperature, relative humidity, and wind velocity) and air pollution (i.e., PM_10_, SO_2_, O_3_, CO, and NO_2_) on HFMD incidence. The current study also aimed to identify potential modifiers and evaluate the modification effects of long-term air pollution levels.

## Methods

### Research location

**Fig. 1:**
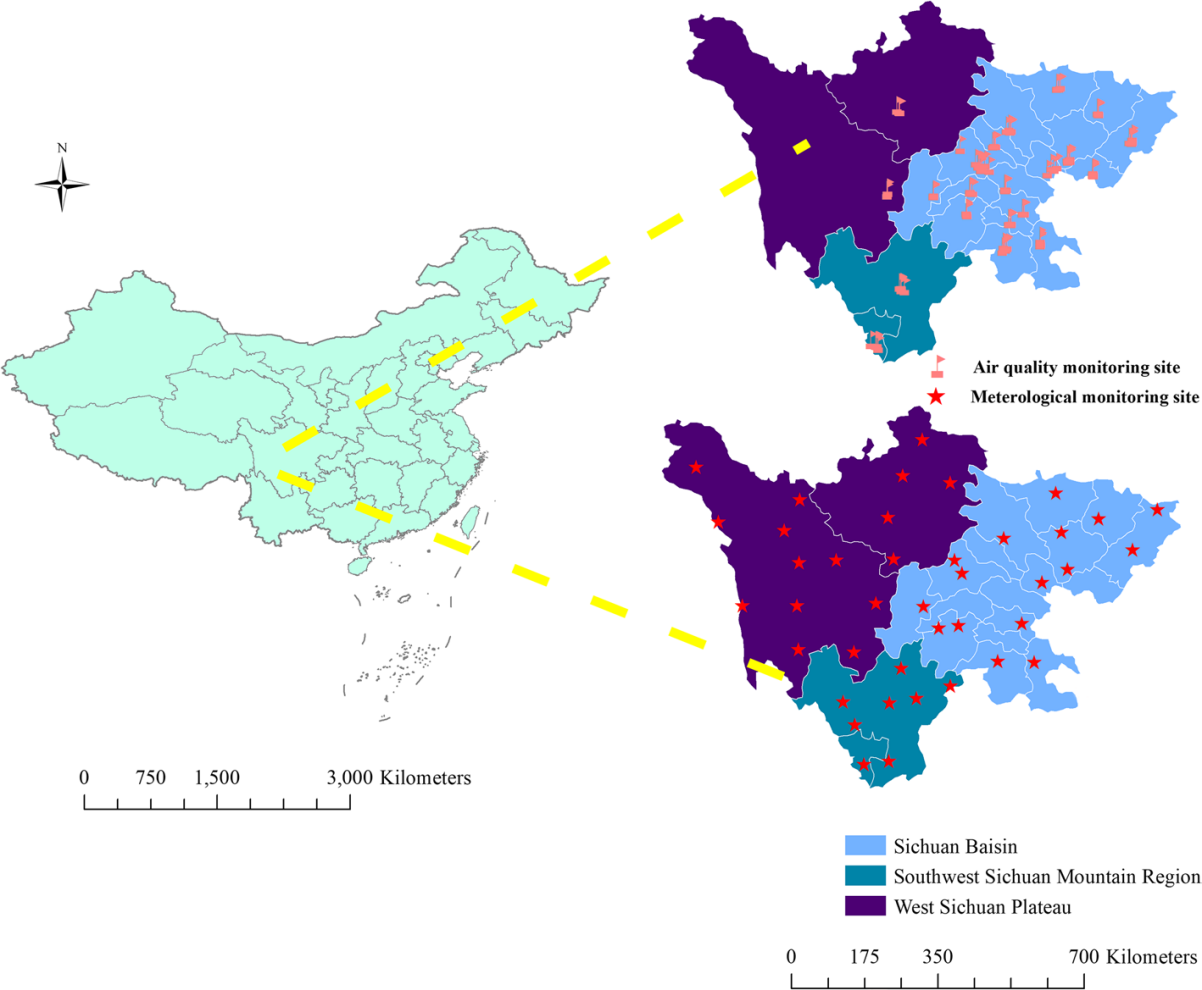
Geographic regions, meteorological monitoring stations and air quality monitoring sites in Sichuan Province; the latter two sets of locations are presented on the division map

Sichuan Province, an inland region in Southwest China, has exhibited continuous increases in population each year since 2005; currently, it contains more than 80 million inhabitants. This province covers approximately 486,000 km^2^ and has 21 prefecture-level cities. The region’s complex topography consists of a basin in the eastern region, a plateau in the western region and a mountainous area in the southwestern region (Fig. 1). The Sichuan Basin, one of the four largest basins in China, has a dense population and a relatively high level of economic development, while western Sichuan is mostly mountainous and sparsely populated. Additionally, there are obvious regional differences in climate. The Sichuan Basin is situated in a subtropical humid monsoon climate zone, in which the average annual temperature is 16–18 ℃ and the average annual precipitation is 1,000–1,300 mm. In addition, the province contains a subhumid subtropical region and an alpine region in the northwest plateau, with long sunshine durations and little annual precipitation. The high levels of emissions, special terrain, and unique climate conditions have resulted in severe air pollution in winter, especially over the eastern basin. The Sichuan Basin accounts for less than 2.7% of the area of China. However, the total emissions of SO_2_, PM_2.5_, and NOx account for 12.1, 8.3, and 5.8% of China’s total emissions, respectively [26].

### Data sources

On May 2, 2008, the Chinese Ministry of Health included HFMD in the catalog of class C notifiable infectious diseases. Information reporting and case management should be carried out within 24 h according to the standard guidelines [27]. The demographic information and illness-related information of each case in 21 prefecture-level cities in Sichuan Province between 2015 and 2017 were collected from the Reporting System of the Chinese Center for Disease Control and Prevention. We extracted the dates that cases presented with symptoms and aggregated them into the daily HFMD counts for the respective 21 cities in Sichuan Province. According to the preliminary analysis, over 99% of reported HFMD patients were less than 15 years old; therefore, this study included only data from patients under the age of 15 years.

We retrieved daily meteorological monitoring data, including ambient temperature (°C) (mean, minimum, and maximum), mean relative humidity (%), mean wind speed (m/s), total sunshine duration (h), and mean atmospheric pressure (hPa) data, from the China Meteorological Data Sharing Service System. Daily surveillance data for air pollution, namely, SO_2_ (μg/m^3^), NO_2_ (μg/m^3^), PM_2.5_ (μg/m^3^), PM_10_ (μg/m^3^), the air quality index (AQI), CO (mg/m^3^), and O_3_ (μg/m^3^) data, were retrieved from the Sichuan Environmental Monitoring Center. The spatial distributions of the meteorological monitoring stations and air quality monitoring sites are shown in Fig. 1. Considering the nature of climatic and air pollution time-series data, a very small proportion of missing values (< 0.1%) were replaced with zeroes for sunshine hours and linear interpolation for other variables. Following the basic principle of matching data from sites closest to the city center, daily HFMD counts, daily meteorological monitoring data, and daily air pollution data were then matched by city-specific codes.

Additionally, we obtained city-level economic (gross domestic product (GDP) per capita), demographic (population density, population growth and number of primary school students), traffic (travel passengers) and health resource characteristics (number of health institutions, number of hospital beds and number of registered physicians) from the Sichuan Statistical Yearbooks during 2015–2017.

We calculated the arithmetic average of each air pollution variable and socioeconomic indicator in the prefecture-level cities as the unit for the whole research period. Then, the arithmetic average of the same variable for each city was designated as a new substitutive variable, denoting the differences in air quality or socioeconomic characteristics among cities. These city-specific variables were included as potential modifiers in the second-stage meta-regression models to assess the modification effects of long-term air pollution levels, which is also known as the explanation of the heterogeneity [28].

### Statistical analysis

In this study, daily HFMD counts, meteorological and air pollution surveillance data from 21 cities in Sichuan Province between 2015 and 2017 were collected, and a multicity time-series approach with two stages was adopted. Epidemiological studies have demonstrated that meteorological and air pollution variables may exhibit complex nonlinear associations with HFMD incidence. To accurately capture the short-term effects of these variables, a unified DLNM with the same modeling structure and parameter determination was first applied for each prefecture-level city to simultaneously quantify the city-specific effect estimates of the exposure and lag dimensions. Then, multivariate meta-regression was performed to combine city-specific effect estimates, and air pollution variables at the city level were fitted as a meta-predictor into models to examine potential modification effects.

#### First-stage analysis

A DLNM based on a quasi-Poisson distribution served as the basic model for detecting possible delayed effects and nonlinear associations between exposures and HFMD incidence for each city in the first stage of analysis [29]. We constructed univariate models to screen for significant associations of meteorological variables and air pollutants with HFMD cases. Then, these significant variables were incorporated into the subsequent multivariate model. Parameters and modeling components were determined on the basis of prior knowledge and a systematic sensitivity analysis strategy [10]. We constructed the final model as follows:

$$\begin{array}{l}{Y}_{t}\sim Quasi-Poisson\left({\mu }_{t}\right)\\ \mathrm{log}\left({\mu }_{t}\right)=\alpha +\sum cb\left(Weather/air pollution, lag\right)+ns\left(Day,df\right)+Auto+Dow+Hod\end{array}$$

where *Y*_*t*_ denotes the daily number of HFMD cases at time *t* in a prefecture-level city and *cb*(*Weather / air pollution, lag*) stands for a cross-basis function of each of the climatic variables and air pollutants that describes the exposure-lag-response relationship. We defined 3 degrees of freedom (dfs) for the natural cubic splines of temperature, relative humidity, wind speed, NO_2_, PM_10_, and ozone in the exposure dimensions, as well as 4 dfs for delayed effects. Considering the incubation period (3–5 days) and the infectious period (nearly 2 weeks) of HFMD [27], the lag range was set to 0–17 days to adequately cover delayed effects. Long-term and seasonal trends in HFMD were eliminated using 8 dfs/year for the natural cubic spline function. Due to the mechanism by which HFMD is transmitted, first- and second-order lag terms on the logarithmic scale of HFMD counts were incorporated to control for residual autocorrelations and are expressed as *Auto* [30]. The day of the week is represented by *Dow*. *Hod* is a binary variable that controls for the effect of national public holidays. The median value of each exposure was assigned as the reference to calculate the relative risks.

#### Second-stage analysis

The cumulative exposure–response relationship in each city was obtained by combining all lag effects to achieve parametric dimensionality reduction for the second stage of analysis. A multivariate meta-regression model containing only intercepts was constructed to capture the overall pooled exposure–response relationship in Sichuan Province and to examine the heterogeneity of city-specific associations. Then, city-specific long-term air pollutant levels and socioeconomic variables were individually added as meta-predictors to fit the meta-regression models with a single meta-predictor to further estimate the modification effects [31]. Variables with P < 0.2 in the single meta-predictor model were selected as alternative factors for the subsequent multiple meta-predictor analysis. All candidate subsets were considered and the best subset of meta-predictors with the lowest AIC was identified. Quantitative statistical indicators, namely, the results of the likelihood ratio test that assessed whether the meta-predictor had statistical significance and the extent of improvement in the I^2^ statistic that indicated the explicable proportion of residual heterogeneity [28], were computed to evaluate the modification effects of long-term air pollution levels.

In addition, we also performed a subgroup analysis based on the three defined regions (the Sichuan Basin, Southwest Sichuan Mountain region, and West Sichuan Plateau). We pooled the effect estimates for each city by region using a multivariate meta-regression model and then visualized the overall pooled exposure–response curves for each region. Limited by the number of cities in the Southwest Sichuan Mountain Region and West Sichuan Plateau; thus, we further explored the potential modification effects of long-term air pollution levels on the associations between environmental factors and HFMD in only the Sichuan Basin.

Multiple climatic variables and air pollutants were assessed in this study. Considering the strong positive correlations among the mean, minimum, and maximum temperatures and among the PM_10_, PM_2.5_, and AQI as well as the strong negative correlation between the mean temperature and air pressure across all 21 cities, only the mean temperature and PM_10_ were included in the final analysis to avoid collinearity. In addition, a considerable proportion (34.6-54.2%) of the actual zero value for sunshine duration was present in multiple cities due to the special topography of the eastern basin; in the southern region of the basin, the proportions approached or even exceeded 50%. When we constructed DLNMs for these cities, the cross-basis matrixes produced multiple collinear variables. Some of these collinear variables were discarded, and the related parameters could not be estimated [31]. Thus, sunshine duration was not included as an exposure variable in the first-stage analysis to ensure the accuracy of parameter estimations. We carried out all analyses with R software (version 4.0.3), with the package *dlnm* to fit all DLNMs and the package *mvmeta* to conduct all multivariate meta-regression models. We constructed geographic maps using ArcGIS software (version 10.0, authorization number: EFL734321752).

## Results

### Research data characteristics

**Fig. 2:**
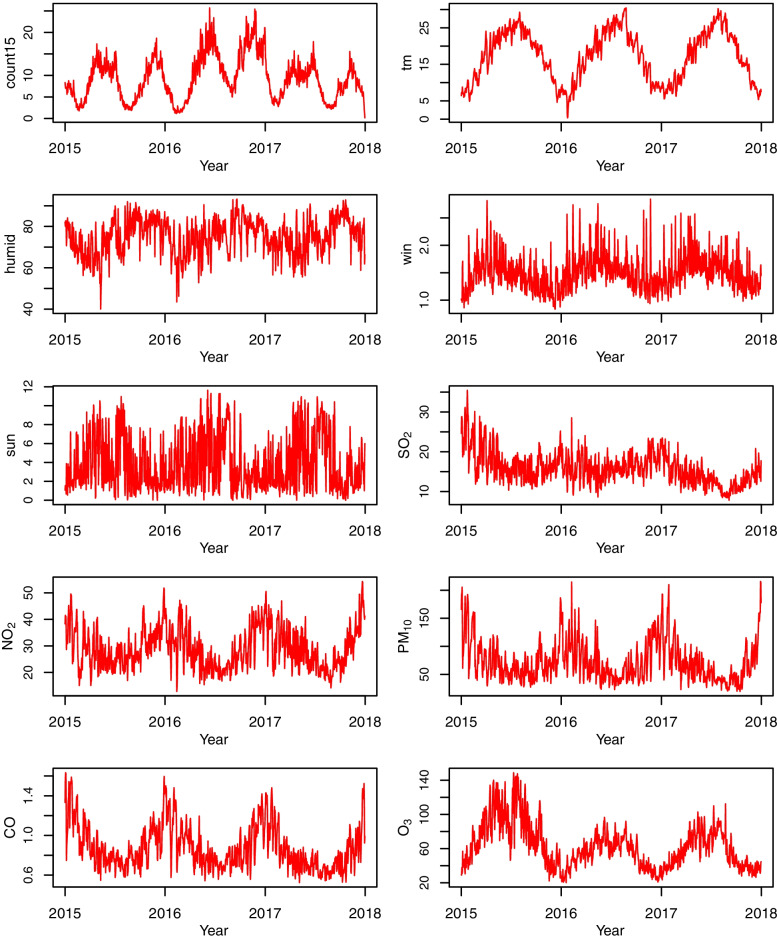
Time series of HFMD cases, four climatic factors, and levels of five air pollutants in Sichuan Province between 2015 and 2017. We calculated the province averages by pooling daily data from 21 cities

A total of 213,973 HFMD cases in children less than 15 years old were recorded in 21 prefecture-level cities in Sichuan Province from 2015 to 2017. The mean annual incidence rate of HFMD was 369.4 cases per 100,000 persons. An average of 9 (range: 0–267) HFMD cases in children were reported each day in the whole study region, with the highest number in Chengdu (79) and the lowest number in Ganzi (1). The daily mean values of climatic factors, such as the mean temperature, relative humidity, wind velocity, and total sunshine hours, were 17.3 °C, 74.9%, 1.5 m/s, and 3.8 h, respectively. The average daily values of SO_2_, NO_2_, PM_10_, CO, and O_3_ levels were 15.8 μg/m^3^, 29.1 μg/m^3^, 72.8 μg/m^3^, 0.9 mg/m^3^, and 62.3 μg/m^3^, respectively (Table 1). The HFMD counts exhibited obvious seasonality with semiannual peaks, one from April to July and the other from September to November. The time-series data of two meteorological factors (i.e., mean temperature and wind speed) and three air pollutants (i.e., NO_2_, PM_10_, and CO) showed similar periodicity and relative stability (Fig. 2).

**Table 1:** Summary statistics of the HFMD counts, meteorological factors, and air pollution for 21 cities in Sichuan Province from 2015 to 2017

Variable	Min	P25	P50	P75	Max	Mean ± SD
Disease counts						
Daily number of HFMD cases	0.0	2.0	4.0	9.0	267.0	9.3 ± 19.6
Meteorological indicators						
Mean temperature (°C)	-10.9	10.8	17.5	23.5	34.1	17.3 ± 7.6
Relative humidity (%)	13.0	67.0	77.0	86.0	100.0	74.9 ± 14.5
Wind speed (m/s)	0.0	1.0	1.4	1.8	6.6	1.5 ± 0.7
Sunshine duration (h)	0.0	0.0	2.7	7.3	13.3	3.8 ± 4.0
Air pollution indicators						
SO_2_ (μg/m^3^)	1.0	9.0	13.0	20.0	117.0	15.8 ± 10.2
NO_2_ (μg/m^3^)	2.0	20.0	27.0	36.0	471.0	29.1 ± 14.0
PM_10_ (μg/m^3^)	3.0	39.0	60.0	93.0	599.0	72.8 ± 47.5
CO (mg/m^3^)	0.1	0.6	0.8	1.1	4.0	0.9 ± 0.4
O_3_ (μg/m^3^)	2.0	38.0	57.0	80.0	243.0	62.3 ± 33.6

### Overall pooled estimates of the relationships of meteorological indicators and air pollutants with HFMD incidence

**Fig. 3:**
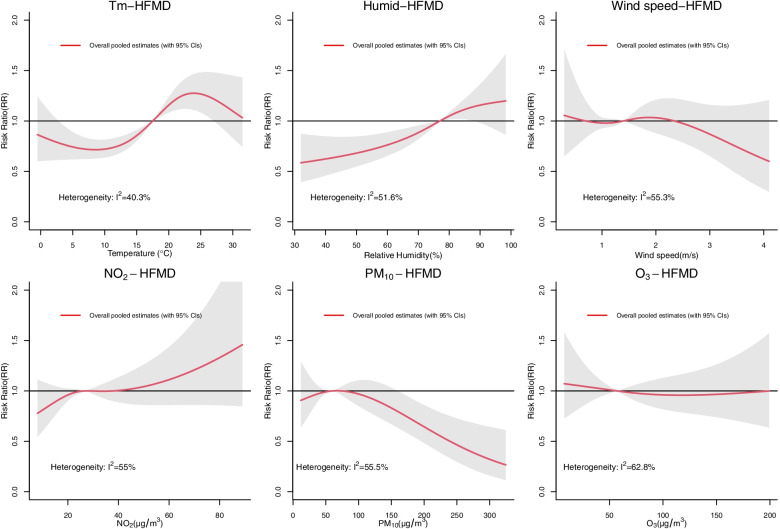
Overall relationship of HFMD risk with meteorological indicators and air pollution variables in Sichuan Province, with the median of each exposure serving as the reference value

The overall pooled cumulative exposure–response relationships of short-term exposure to meteorological indicators and air pollutants with HFMD incidence presented three main patterns. First, the cumulative risk ratio (RR) of HFMD increased with temperature until it peaked at 24 °C (RR = 1.28, 95% CI: 1.11–1.47), and then the curve significantly declined. Once the temperature exceeded 27 °C, there was no significant relationship between temperature and HFMD incidence. Second, with increasing relative humidity, the RR continued to increase in an approximately positive linear pattern, especially in low to moderate relative humidity. Conversely, the short-term impact of PM_10_ on HFMD showed a negative linear pattern at high PM_10_ concentrations. Third, for NO_2_, we found a positive linear relationship with HFMD incidence at both low and high NO_2_ concentrations. Conversely, once the wind speed exceeded the median value, we found a negative linear relationship of HFMD incidence with increasing wind speed (Fig. 3). However, the 95% confidence intervals (CIs) of the relative risks of NO_2_, wind speed, and O_3_ included 1, indicating these relationships were not statistically significant. In our subgroup analysis, we found that the risk of HFMD decreased with increasing O_3_ levels in the Southwest Sichuan Mountain Region and that the relationship between temperature and HFMD incidence was approximately negative and linear in the West Sichuan Plateau (Figs. S1 and S2 in the Supplementary Information).

### Modification effects of long-term air pollution levels

Although we constructed all models for each city with the same set of parameters and components, the city-specific exposure–response relationships showed considerable heterogeneity, with the I^2^ statistic ranging from 40.3% for the relationship between temperature and HFMD incidence to 62.8% for the relationship between O_3_ and HFMD incidence (Fig. 3 and Fig. S4 in the Supplementary Information). Long-term air pollutants were found to significantly modify all the associations between meteorological indicators and HFMD incidence (*P* < 0.05 on the likelihood ratio test). SO_2_ levels modified both the relationship between temperature and HFMD (*P* = 0.039) and the relationship between relative humidity and HFMD (*P* = 0.024). CO levels were also identified exerting substantial modification effects on the association of HFMD incidence with temperature (*P* = 0.027) and wind speed (*P* = 0.044) (Table 2). Among all the single meta-predictor models, we found that a significant modification effect of CO level on the relationship of HFMD with ambient temperature showed the largest improvement in reducing heterogeneity, with ΔI^2^ equal to 5.9%. However, no long-term air pollutant significantly modified any of the relationships between short-term exposure to air pollution and HFMD incidence (see Tables S1, S2, S3, S4, S5 and S6 in the Supplementary Information). We found that the heterogeneity of the relationship between temperature and HFMD incidence was decreased from 40.3 to 27.5% by including both SO_2_ and NO_2_ levels in the model. Moreover, the incorporation of PM_10_ level, CO level, and GDP per capita in the model significantly reduced the heterogeneity of the relationship between wind speed and HFMD incidence, with ΔI^2^ equal to 7% (Table 2). In our subgroup analysis, no significant modification effect was observed on the relationship between temperature and HFMD incidence in the Sichuan Basin (Table S7 in the Supplementary Information).

**Table 2:** Quantitative statistics indicate that long-term air pollution levels significantly modify the relationships of HFMD incidence with short-term exposure to meteorological factors and air pollution, according to the multivariate meta-regression analysis

Exposure	Modifier	LR test			Cochran Q test		Model fit	Heterogeneity	
		Test statistic	df	*P* value	Q	df	AIC	I^2^	ΔI^2^
Mean temperature	SO_2_	8.394	3	0.039	90.742	57	195.572	37.2	-3.1
	CO	9.162	3	0.027	86.873	57	194.804	34.4	-5.9
	SO_2_ + NO_2_	16.809	6	0.010	74.517	54	193.158	27.5	-12.8
	Reference	-	-	-	100.513	60	197.966	40.3	0
Relative humidity	SO_2_	9.481	3	0.024	113.733	57	187.55	49.9	-1.7
	Reference	-	-	-	124.026	60	191.031	51.6	0
Wind speed	CO	8.123	3	0.044	122.983	57	242.044	53.7	-1.6
	PM_10_ + CO + Per_GDP	21.63	9	0.010	98.559	51	240.537	48.3	-7
	Reference	-	-	-	134.348	60	244.167	55.3	0

**Fig. 4:**
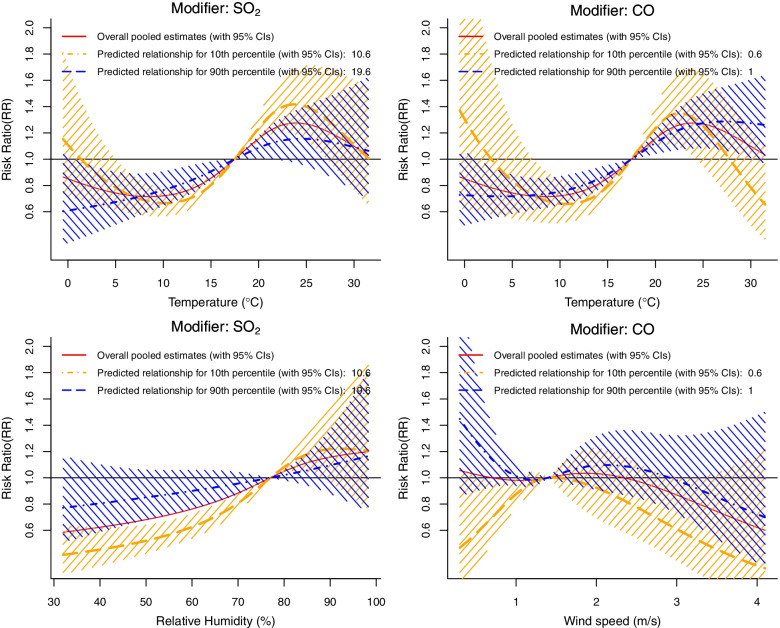
Estimated exposure–response relationships with the significant effect modifiers at relatively low and high levels (i.e., 10^th^ and 90^th^ percentiles)

After visualizing the modification effects of the significant meta-predictors, we further found that SO_2_ and CO levels modified the exposure–response curve of the relationship between temperature and HFMD incidence, mainly at low and high temperatures. We found that the 10^th^ percentiles of SO_2_ and CO concentrations reinforced the effects of temperature, especially cold temperatures, on HFMD risk. In contrast, the 10^th^ percentile of SO_2_ concentrations showed relatively strong protective effects of relative humidity in the left tail (the relative humidity ranged from 32 to 77%). Given the modification effects of SO_2_ level on the relationship between relative humidity and HFMD incidence, we compared the minimum relative humidity (32%) to the median relative humidity (77%); the RR was 0.77 (95% CI: 0.51–1.17) when SO_2_ concentration was in the 90^th^ percentile (19.6 μg/m^3^) and 0.41 (95% CI: 0.27–0.64) when SO_2_ concentration was in the 10^th^ percentile (10.6 μg/m^3^). We further found that CO levels partly modified the shape of the wind speed-HFMD curve. In general, this relationship exhibited approximately inverse N-shaped and inverse U-shaped curves at 90^th^ percentile and 10^th^ percentile of CO concentration, respectively (Fig. 4).

## Discussion

In the present study, we applied a two-stage analysis to characterize the associations of multiple meteorological indicators and air pollutants with HFMD incidence in children, focusing on whether and how long-term air pollution levels modified the associations of short-term exposure to climatic variables and air pollutants with HFMD incidence. To the best of our knowledge, this is the first study to quantify the complex short-term impacts of exposure to multiple air pollutants on HFMD incidence in children with a multicity time-series analysis. Importantly, this study is the first to examine the potential modification effects of long-term air pollution levels on the relationships between environmental variables and HFMD.

Our study revealed that a nonlinear inverted V-shaped pattern described the relationship between temperature and HFMD and that there was a positive correlation between relative humidity and HFMD incidence. Therefore, both high temperature and high relative humidity exert adverse effects on HFMD incidence. These results are consistent with previous studies conducted in other regions. For example, inverted V-shaped patterns that describe the relationship between temperature and HFMD have been reported for Guangdong [32], Wuhan [33], and Beijing [34], and a similar positive association between relative humidity and HFMD has been reported in Southwest China [24]. Host activity, enterovirus excretion, and the survival time of enteroviruses in vitro might be the important pathways by which meteorological factors affect HFMD incidence.

Among the relationships between air pollutants and HFMD, we found an approximately linear relationship between PM_10_ levels and HFMD. Compared with moderate PM_10_ concentrations, the risks of HFMD decreased at higher PM_10_ concentrations. Several previously proposed theories may explain this phenomenon. Particulate matter is conducive to the attachment of enteroviruses and the transmission of HFMD [35, 36]. Moreover, particulate matter tends to increase inflammation and therefore exacerbate the susceptibility to viral infectious diseases [37]. Besides, susceptible people are prone to reduce their outdoor activities or wear masks to protect themselves on high-air pollution days [38]. However, a study in Ningbo [17] and a study in Shenzhen [20] both showed that short-term exposure to PM_10_ was not significantly associated with HFMD incidence, in contrast with our results. Ningbo and Shenzhen are typical coastal cities in mainland China, while Sichuan is an inland province. These regions exhibit obvious discrepancies in their economies, climates, and lifestyles. Furthermore, the Sichuan Basin is a heavily polluted area with much higher particulate air pollution than other regions; therefore, we propose that the short-term effects of PM_10_ on HFMD are more likely to be identified in this area. In such a heavily polluted area, which is characterized by exposure to high levels of particulate matter, the impact of PM_10_ on HFMD incidence cannot be ignored, especially at the medium-range scale.

Although the same set of parameters and components were used to construct the DLNM for each prefecture-level city to eliminate heterogeneity in the model specifications, our results suggest that a moderate proportion of heterogeneity is due to true differences among regions in the short-term impacts of meteorological variables and air pollutants on HFMD. We found that CO and SO_2_ levels were important effect modifiers of this relationship that explained some of the heterogeneity. High concentrations of CO and SO_2_ reduced the risk of HFMD at low temperatures. Reduced enterovirus activity at low temperatures may decrease the chance of transmission to hosts [39]. Similarly, self-protection measures, including wearing masks and reducing outdoor activities during periods of high concentrations of CO and SO_2_, could also decrease exposure in susceptible children [38]; therefore, air pollution reinforces the protective effects of low temperatures on HFMD. In addition, we found that the risk of HFMD at high temperatures was enhanced by high CO concentrations. Previous studies have indicated that CO exposure exerts several adverse health impacts, such as inflammation and oxidative damage [40, 41]. Therefore, an increased risk of HFMD was observed with severe CO exposure. However, after excluding the plateau and mountain regions, we did not find that CO levels significantly modified the relationship between temperature and HFMD. This implies that targeted public health measures considering both direct and indirect pathway of CO and SO_2_ on HFMD could reduce the health impacts of temperature according to the specific relationships between temperature and HFMD in the two regions.

In addition to the modification effects of long-term air pollution levels on the association between temperature and HFMD, our results also suggested that high SO_2_ concentrations reduced the influence of relative humidity on HFMD, especially when the relative humidity was below the median level. Due to the high water solubility of SO_2_, these pollutants are more likely to enter and damage the mucosa of the upper respiratory tract [42, 43]. The respiratory tract is one of the most important transmission routes of HFMD. Thus, we can reasonably speculate that SO_2_ invasion in the body under humid conditions will increase susceptibility to HFMD. Additionally, Sichuan Province consists of complex and unique terrain, with high humidity experienced year-round, especially in the eastern basin. Therefore, even under conditions of low and moderate humidity, this phenomenon may be easily observed. However, SO_2_ is consumed under higher air-humidity conditions, and the modification effects of SO_2_ on the relationship between high relative humidity and HFMD weakens and becomes nonsignificant under such conditions. In addition, at low wind speeds, the relationship between wind speed and HFMD shifted from a positive linear relationship to a generally negative linear relationship in the presence of low and high CO levels, respectively. On low-air pollution days with a wind speed within the comfortable range, children are likely to participate in outdoor activities; wind will accelerate the spread of enteroviruses through airborne droplets [44, 45]. The complex impacts of various air pollution indicators and meteorological factors on HFMD are generally caused by several underlying mechanisms, which have yet to be revealed. In summary, these findings suggest that in areas exposed to different long-term concentrations of CO and SO_2_, public health policies (e.g., reducing ambient air pollution, increasing mask wearing and staying indoors) should consider the modification effects of long-term air pollution levels to reduce the adverse health impacts of meteorological factors on HFMD incidence.

Although this study provides new insights into the short-term effects of environmental factors on HFMD incidence and the potential modification effects of long-term air pollution levels, several limitations should be noted. First, due to the nature of this ecological study, we obtained associations at the population level, which limits causal inference. Second, a considerable part of the heterogeneity was not explained by air pollutant levels. Other variables, such as public health interventions, individual self-protection measures, and customs, may also be potential modifiers; however, these variables are difficult to explore due to the lack of data accessibility. Our results indicate that air pollution also plays a major part in the relationships between environmental factors and HFMD as an effect modifier. These results are highly important for determining the combined effects of multiple factors to formulate more sophisticated and effective public health strategies to protect vulnerable populations from HFMD.

## Conclusion

In conclusion, the present study demonstrates that the short-term effects of meteorological factors on HFMD incidence were modified by long-term SO_2_ and CO levels in Sichuan Province. Therefore, the interactions between meteorological factors and air pollutant levels should be considered when assessing the health effects of these environmental factors on the incidence of HFMD. The modification effects of long-term air pollution exposure on the relationships between HFMD incidence and meteorological factors can inform health authorities to optimize public health policies including reducing ambient air pollution and reinforcing self-protective actions among vulnerable children. Our findings also extend the previously limited findings in heavily polluted areas and enhance current understanding of the short-term associations between meteorological factors and HFMD incidence.

## Supplementary Information

**Additional file 1: Fig. S1. **Overall relationship of HFMD counts with meteorological factors and air pollution in the Southwest Sichuan Mountain Region. **Fig. S2. **Overall relationship of HFMD counts with meteorological factors and air pollution in the West Sichuan Plateau. **Fig. S3.** Overall relationship of HFMD counts with meteorological factors and air pollution in the Sichuan Basin. **Fig. S4.** City-specific relationship of HFMD counts with meteorological factors and air pollution in 21 prefecture-level cities in Sichuan Province. **Table S1.** Multivariate meta-regression models for the relationships between temperature and HFMD. **Table S2**. Multivariate meta-regression models for the relationships between relative humidity and HFMD. **Table S3**. Multivariate meta-regression models for the relationships between wind speed and HFMD. **Table S4**. Multivariate meta-regression models for the relationships between NO_2_ and HFMD. **Table S5**. Multivariate meta-regression models for the relationships between PM_10_ and HFMD. **Table S6**. Multivariate meta-regression models for the relationships between O_3_ and HFMD. **Table S7.** Significant effect modifiers of long-term air pollution indicators on the relationship of HFMD with short-term exposure to meteorological factors and air pollution according to the multivariate meta-regression analysis in the Sichuan Basin.

## Data Availability

The data that generated during and analyzed during the current study are available from Sichuan CDC but restrictions apply to the availability of these data, which were used under license for the current study, and so are not publicly available. But data are available from the corresponding author and with permission of Sichuan CDC on reasonable request.

## Abbreviations

HFMD: Hand, foot, and mouth disease
DLNM: Distributed lag nonlinear model
RR: Relative risk
CI: Confidence interval
