# Supplementary material for: Long-term air pollution levels modify the relationships between short-term exposure to meteorological factors, air pollution and the incidence of hand, foot and mouth disease in children: a DLNM-based multicity time series study in Sichuan Province, China
Source: BMC Public Health. 2022 Aug 4;22:1484. doi: 10.1186/s12889-022-13890-7 (PMC9351082; doi:10.1186/s12889-022-13890-7)
Supplement: Supplementary file 1 — Additional file 1: Fig. S1. Overall relationship of HFMD counts with meteorological factors and air pollution in the Southwest Sichuan Mountain Region. Fig. S2. Overall relationship of HFMD counts with meteorological factors and air pollution in the West Sichuan Plateau. Fig. S3. Overall relationship of HFMD counts with meteorological factors and air pollution in the Sichuan Basin. Fig. S4. City-specific relationship of HFMD counts with meteorological factors and air pollution in 21 prefecture-level cities in Sichuan Province. Table S1. Multivariate meta-regression models for the relationships between temperature and HFMD. Table S2. Multivariate meta-regression models for the relationships between relative humidity and HFMD. Table S3. Multivariate meta-regression models for the relationships between wind speed and HFMD. Table S4. Multivariate meta-regression models for the relationships between NO2 and HFMD. Table S5. Multivariate meta-regression models for the relationships between PM10 and HFMD. Table S6. Multivariate meta-regression models for the relationships between O3 and HFMD. Table S7. Significant effect modifiers of long-term air pollution indicators on the relationship of HFMD with short-term exposure to meteorological factors and air pollution according to the multivariate meta-regression analysis in the Sichuan Basin. [file 12889_2022_13890_MOESM1_ESM.docx]

**Long-term air pollution levels modify the relationships between short-term exposure to meteorological factors, air pollution and the incidence of hand, foot and mouth disease in children: A DLNM-based multicity time series study in Sichuan Province, China**

**Figures**

**Fig. S1. Overall relationship of HFMD counts with meteorological factors and air pollution in the Southwest Sichuan Mountain Region.**

**Fig. S2. Overall relationship of HFMD counts with meteorological factors and air pollution in the West Sichuan Plateau.**

**Fig. S3. Overall relationship of HFMD counts with meteorological factors and air pollution in the Sichuan Basin.**

**Fig. S4. City-specific relationship of HFMD counts with meteorological factors and air pollution in 21 prefecture-level cities in Sichuan Province.**

**Tables**

**Table S1 Multivariate meta-regression models for the relationships between temperature and HFMD.**

**Table S2 Multivariate meta-regression models for the relationships between relative humidity and HFMD.**

**Table S3 Multivariate meta-regression models for the relationships between wind speed and HFMD.**

**Table S4 Multivariate meta-regression models for the relationships between NO_2_ and HFMD.**

**Table S5 Multivariate meta-regression models for the relationships between PM_10_ and HFMD.**

**Table S6 Multivariate meta-regression models for the relationships between O_3_ and HFMD.**

**Table S7 Significant effect modifiers of long-term air pollution indicators on the relationship of HFMD with short-term exposure to meteorological factors and air pollution according to the multivariate meta-regression analysis in the Sichuan Basin.**


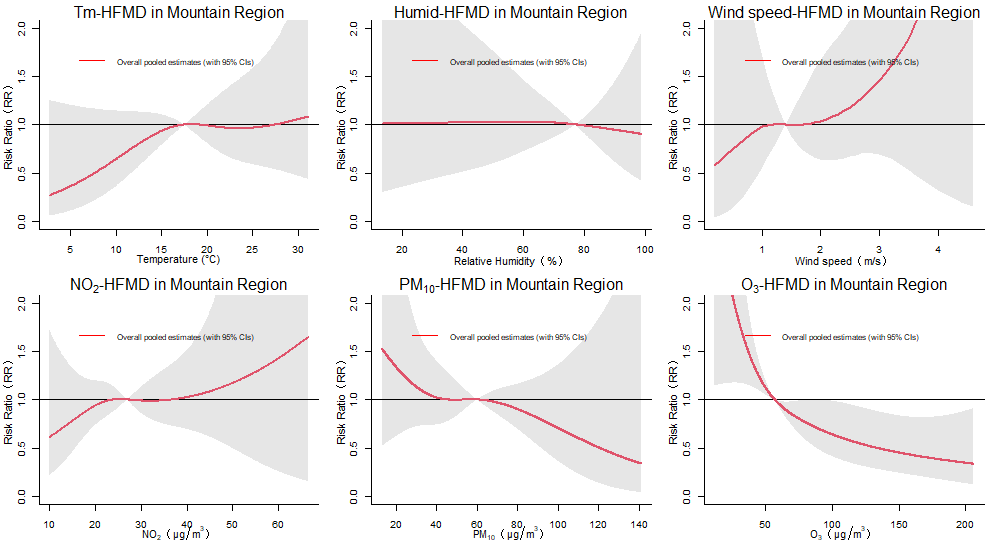


Fig. S1. Overall relationship of HFMD counts with meteorological factors and air pollution in the Southwest Sichuan Mountain Region.


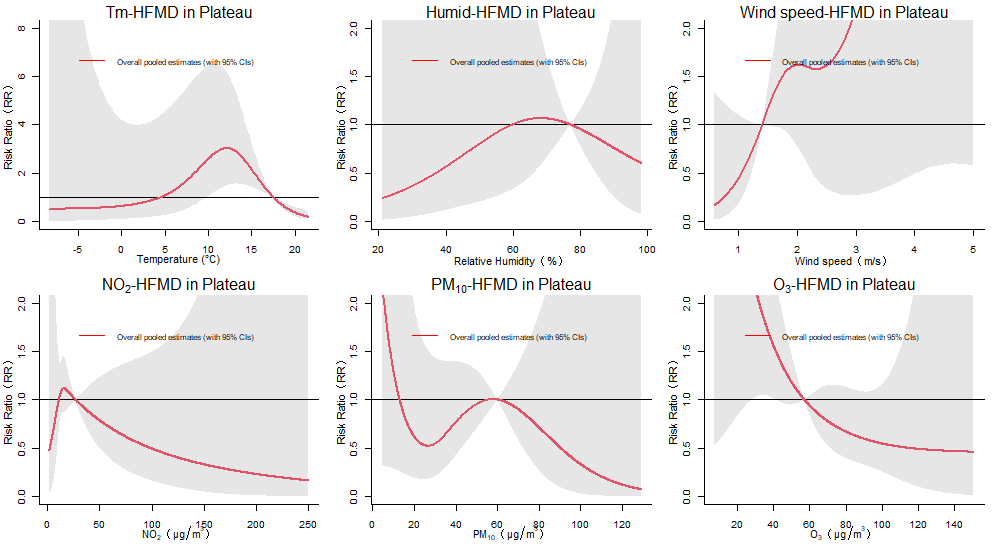


Fig. S2. Overall relationship of HFMD counts with meteorological factors and air pollution in the West Sichuan Plateau.


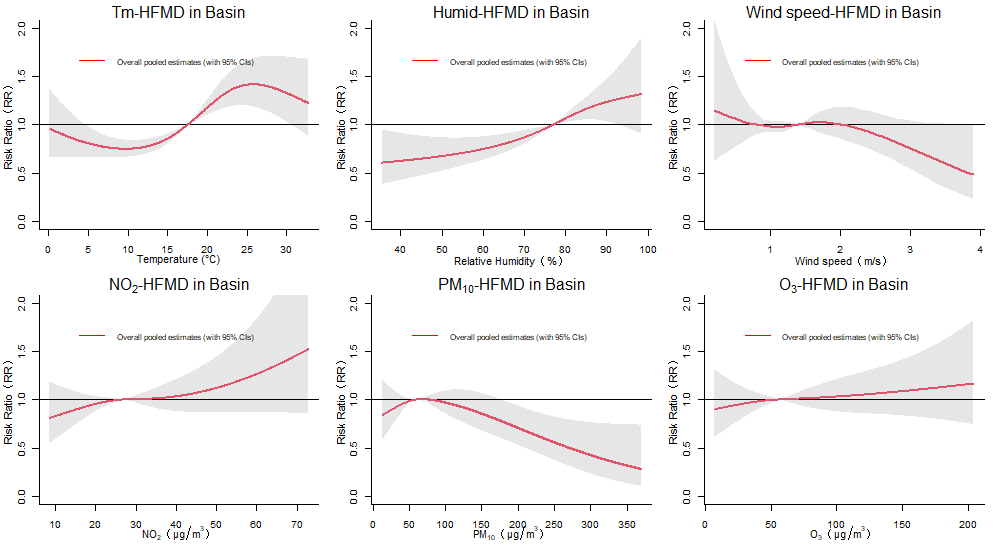


Fig. S3. Overall relationship of HFMD counts with meteorological factors and air pollution in the Sichuan Basin.


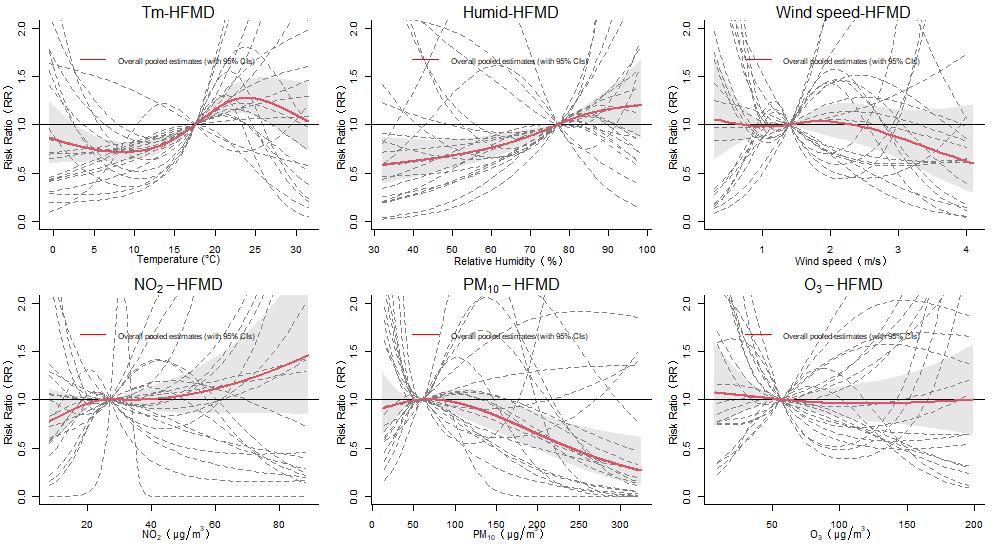


Fig. S4. City-specific relationship of HFMD counts with meteorological factors and air pollution in 21 prefecture-level cities in Sichuan Province.

Table S1 Multivariate meta-regression models for the relationships between temperature and HFMD.

| Modifier | LR test | | | | Cochran Q test | | Model fit | Heterogeneity | |  |
| --- | --- | --- | --- | --- | --- | --- | --- | --- | --- | --- |
|  | stats | df | *P* value | | Q | df | AIC | I^2^ | ΔI^2^ | |
| Single meta-predictor models | | | |  |  |  |  |  |  | |
| SO_2_ | 8.394 | 3 | | 0.039 | 90.742 | 57 | 195.572 | 37.2 | -3.1 | |
| NO_2_ | 4.987 | 3 | | 0.173 | 90.024 | 57 | 198.979 | 36.7 | -3.6 | |
| PM_10_ | 2.831 | 3 | | 0.418 | 93.504 | 57 | 201.135 | 39 | -1.3 | |
| CO | 9.162 | 3 | | 0.027 | 86.873 | 57 | 194.804 | 34.4 | -5.9 | |
| O_3_ | 0.933 | 3 | | 0.817 | 98.65 | 57 | 203.033 | 42.2 | 1.9 | |
| Per_GDP | 6.95 | 3 | | 0.074 | 87.799 | 57 | 197.016 | 35.1 | -5.2 | |
| Pop_den | 0.887 | 3 | | 0.828 | 98.005 | 57 | 203.079 | 41.8 | 1.5 | |
| Pop_Growth | 3.603 | 3 | | 0.308 | 91.909 | 57 | 200.364 | 38 | -2.3 | |
| Students | 1.409 | 3 | | 0.703 | 93.682 | 57 | 202.557 | 39.2 | -1.1 | |
| Passengers | 0.265 | 3 | | 0.966 | 96.103 | 57 | 203.701 | 40.7 | 0.4 | |
| Health_insiti | 0.545 | 3 | | 0.909 | 96.859 | 57 | 203.421 | 41.2 | 0.9 | |
| Health_bed | 0.904 | 3 | | 0.824 | 94.885 | 57 | 203.062 | 39.9 | -0.4 | |
| Health_physi | 0.925 | 3 | | 0.819 | 94.665 | 57 | 203.041 | 39.8 | -0.5 | |
| reference | - | - | | - | 100.513 | 60 | 197.966 | 40.3 | 0 | |
| Multiple meta-predictors model | | | |  |  |  |  |  |  | |
| SO_2_+NO_2_ | 16.809 | 6 | | 0.010 | 74.517 | 54 | 193.158 | 27.5 | -12.8 | |

Table S2 Multivariate meta-regression models for the relationships between relative humidity and HFMD.

| Modifier | LR test | | | | Cochran Q test | | Model fit | Heterogeneity | |  |
| --- | --- | --- | --- | --- | --- | --- | --- | --- | --- | --- |
|  | stats | df | *P* value | | Q | df | AIC | I^2^ | ΔI^2^ | |
| Single meta-predictor models | | | |  |  |  |  |  |  | |
| SO_2_ | 9.481 | 3 | | 0.024 | 113.733 | 57 | 187.55 | 49.9 | -1.7 | |
| NO_2_ | 3.484 | 3 | | 0.323 | 107.83 | 57 | 193.547 | 47.1 | -4.5 | |
| PM_10_ | 2.001 | 3 | | 0.572 | 114.244 | 57 | 195.03 | 50.1 | -1.5 | |
| CO | 0.417 | 3 | | 0.937 | 120.686 | 57 | 196.614 | 52.8 | 1.2 | |
| O_3_ | 0.098 | 3 | | 0.992 | 123.689 | 57 | 196.933 | 53.9 | 2.3 | |
| Per_GDP | 4.617 | 3 | | 0.202 | 108.837 | 57 | 192.414 | 47.6 | -4 | |
| Pop_den | 0.888 | 3 | | 0.828 | 117.737 | 57 | 196.143 | 51.6 | 0 | |
| Pop_Growth | 0.056 | 3 | | 0.997 | 122.986 | 57 | 196.975 | 53.7 | 2.1 | |
| Students | 0.79 | 3 | | 0.852 | 116.131 | 57 | 196.241 | 50.9 | -0.7 | |
| Passengers | 1.159 | 3 | | 0.763 | 114.237 | 57 | 195.872 | 50.1 | -1.5 | |
| Health_insiti | 1.699 | 3 | | 0.637 | 120.347 | 57 | 195.332 | 52.6 | 1 | |
| Health_bed | 0.736 | 3 | | 0.865 | 115.921 | 57 | 196.295 | 50.8 | -0.8 | |
| Health_physi | 1.144 | 3 | | 0.767 | 114.633 | 57 | 195.887 | 50.3 | -1.3 | |
| reference | - | - | | - | 124.026 | 60 | 191.031 | 51.6 | 0 | |
| Multiple meta-predictors model | | | | |  |  |  |  |  | |
| - | - | - | | - | - | - | - | - | - | |

Table S3 Multivariate meta-regression models for the relationships between wind speed and HFMD.

| Modifier | LR test | | | | Cochran Q test | | Model fit | Heterogeneity | |  |
| --- | --- | --- | --- | --- | --- | --- | --- | --- | --- | --- |
|  | stats | df | *P* value | | Q | df | AIC | I^2^ | ΔI^2^ | |
| Single meta-predictor models | | | |  |  |  |  |  |  | |
| SO_2_ | 4.641 | 3 | | 0.2 | 126.574 | 57 | 245.526 | 55 | -0.3 | |
| NO_2_ | 3.879 | 3 | | 0.275 | 129.057 | 57 | 246.288 | 55.8 | 0.5 | |
| PM_10_ | 5.762 | 3 | | 0.124 | 131.523 | 57 | 244.405 | 56.7 | 1.4 | |
| CO | 8.123 | 3 | | 0.044 | 122.983 | 57 | 242.044 | 53.7 | -1.6 | |
| O_3_ | 2.837 | 3 | | 0.417 | 128.031 | 57 | 247.33 | 55.5 | 0.2 | |
| Per_GDP | 5.816 | 3 | | 0.121 | 121.459 | 57 | 244.35 | 53.1 | -2.2 | |
| Pop_den | 2.028 | 3 | | 0.567 | 128.21 | 57 | 248.139 | 55.5 | 0.2 | |
| Pop_Growth | 6.843 | 3 | | 0.077 | 123.361 | 57 | 243.323 | 53.8 | -1.5 | |
| Students | 0.442 | 3 | | 0.931 | 129.698 | 57 | 249.725 | 56.1 | 0.8 | |
| Passengers | 0.616 | 3 | | 0.893 | 128.356 | 57 | 249.551 | 55.6 | 0.3 | |
| Health_insiti | 0.412 | 3 | | 0.938 | 131.577 | 57 | 249.755 | 56.7 | 1.4 | |
| Health_bed | 0.83 | 3 | | 0.842 | 127.659 | 57 | 249.337 | 55.3 | 0 | |
| Health_physi | 1.423 | 3 | | 0.7 | 126.615 | 57 | 248.744 | 55 | -0.3 | |
| reference | - | - | | - | 134.348 | 60 | 244.167 | 55.3 | 0 | |
| Multiple meta-predictors model | | | |  |  |  |  |  |  | |
| PM_10_+CO+ Per_GDP | 21.63 | 9 | | 0.010 | 98.559 | 51 | 240.537 | 48.3 | -7 | |

Table S4 Multivariate meta-regression models for the relationships between NO_2_ and HFMD.

| Modifier | LR test | | | | Cochran Q test | | Model fit | Heterogeneity | |  |
| --- | --- | --- | --- | --- | --- | --- | --- | --- | --- | --- |
|  | stats | df | *P* value | | Q | df | AIC | I^2^ | ΔI^2^ | |
| Single meta-predictor models | | | |  |  |  |  |  |  | |
| SO_2_ | 3.122 | 3 | | 0.373 | 129.791 | 57 | 231.101 | 56.1 | 1.1 | |
| PM_10_ | 0.781 | 3 | | 0.854 | 133.01 | 57 | 233.441 | 57.1 | 2.1 | |
| CO | 0.599 | 3 | | 0.897 | 132.587 | 57 | 233.624 | 57 | 2 | |
| O_3_ | 4.047 | 3 | | 0.256 | 123.992 | 57 | 230.176 | 54 | -1 | |
| Per_GDP | 0.233 | 3 | | 0.972 | 133.191 | 57 | 233.990 | 57.2 | 2.2 | |
| Pop_den | 1.329 | 3 | | 0.722 | 132.52 | 57 | 232.894 | 57 | 2 | |
| Pop_Growth | 1.498 | 3 | | 0.683 | 127.916 | 57 | 232.725 | 55.4 | 0.4 | |
| Students | 0.91 | 3 | | 0.823 | 131.035 | 57 | 233.313 | 56.5 | 1.5 | |
| Passengers | 0.656 | 3 | | 0.884 | 131.641 | 57 | 233.567 | 56.7 | 1.7 | |
| Health_insiti | 0.45 | 3 | | 0.93 | 131.358 | 57 | 233.772 | 56.6 | 1.6 | |
| Health_bed | 0.28 | 3 | | 0.964 | 131.88 | 57 | 233.943 | 56.8 | 1.8 | |
| Health_physi | 0.243 | 3 | | 0.97 | 131.949 | 57 | 233.98 | 56.8 | 1.8 | |
| reference | - | - | | - | 133.308 | 60 | 228.223 | 55 | 0 | |
| Multiple meta-predictors model | | | |  |  |  |  |  |  | |
| - | - | - | | - | - | - | - | - | - | |

Table S5 Multivariate meta-regression models for the relationships between PM_10_ and HFMD.

| Modifier | LR test | | | | Cochran Q test | | Model fit | Heterogeneity | |  |
| --- | --- | --- | --- | --- | --- | --- | --- | --- | --- | --- |
|  | stats | df | *P* value | | Q | df | AIC | I^2^ | ΔI^2^ | |
| Single meta-predictor models | | | |  |  |  |  |  |  | |
| SO_2_ | 0.143 | 3 | | 0.986 | 134.555 | 57 | 238.797 | 57.6 | 2.1 | |
| NO_2_ | 5.35 | 3 | | 0.148 | 130.665 | 57 | 233.591 | 56.4 | 0.9 | |
| CO | 1.147 | 3 | | 0.766 | 132.553 | 57 | 237.793 | 57 | 1.5 | |
| O_3_ | 3.639 | 3 | | 0.303 | 122.712 | 57 | 235.302 | 53.5 | -2 | |
| Per_GDP | 1.038 | 3 | | 0.792 | 131.5 | 57 | 237.902 | 56.7 | 1.2 | |
| Pop_den | 0.51 | 3 | | 0.917 | 130.425 | 57 | 238.431 | 56.3 | 0.8 | |
| Pop_Growth | 1.226 | 3 | | 0.747 | 131.676 | 57 | 237.714 | 56.7 | 1.2 | |
| Students | 1.258 | 3 | | 0.739 | 128.992 | 57 | 237.682 | 55.8 | 0.3 | |
| Passengers | 0.572 | 3 | | 0.903 | 130.021 | 57 | 238.368 | 56.2 | 0.7 | |
| Health_insiti | 2.932 | 3 | | 0.402 | 124.961 | 57 | 236.009 | 54.4 | -1.1 | |
| Health_bed | 1.281 | 3 | | 0.734 | 129.4 | 57 | 237.659 | 56 | 0.5 | |
| Health_physi | 1.006 | 3 | | 0.8 | 130.18 | 57 | 237.934 | 56.2 | 0.7 | |
| reference | - | - | | - | 134.783 | 60 | 232.94 | 55.5 | 0 | |
| Multiple meta-predictors model | | | |  |  |  |  |  |  | |
| - | - | - | | - | - | - | - | - | - | |

Table S6 Multivariate meta-regression models for the relationships between O_3_ and HFMD.

| Modifier | LR test | | | | Cochran Q test | | Model fit | Heterogeneity | |  |
| --- | --- | --- | --- | --- | --- | --- | --- | --- | --- | --- |
|  | stats | df | *P* value | | Q | df | AIC | I^2^ | ΔI^2^ | |
| Single meta-predictor models | | | |  |  |  |  |  |  | |
| SO_2_ | 4.706 | 3 | | 0.195 | 151.926 | 57 | 209.86 | 62.5 | -0.3 | |
| NO_2_ | 0.376 | 3 | | 0.945 | 154.239 | 57 | 214.19 | 63 | 0.2 | |
| PM_10_ | 2.38 | 3 | | 0.497 | 160.432 | 57 | 212.186 | 64.5 | 1.7 | |
| CO | 0.066 | 3 | | 0.996 | 159.191 | 57 | 214.5 | 64.2 | 1.4 | |
| Per_GDP | 3.355 | 3 | | 0.34 | 146.92 | 57 | 211.211 | 61.2 | -1.6 | |
| Pop_den | 0.13 | 3 | | 0.988 | 153.139 | 57 | 214.436 | 62.8 | 0 | |
| Pop_Growth | 6.684 | 3 | | 0.083 | 143.975 | 57 | 207.882 | 60.4 | -2.4 | |
| Students | 3.014 | 3 | | 0.389 | 151.247 | 57 | 211.552 | 62.3 | -0.5 | |
| Passengers | 0.769 | 3 | | 0.857 | 152.401 | 57 | 213.797 | 62.6 | -0.2 | |
| Health_insiti | 2.496 | 3 | | 0.476 | 154.46 | 57 | 212.07 | 63.1 | 0.3 | |
| Health_bed | 0.174 | 3 | | 0.982 | 154.016 | 57 | 214.392 | 63 | 0.2 | |
| Health_physi | 0.309 | 3 | | 0.958 | 152.931 | 57 | 214.257 | 62.7 | -0.1 | |
| reference | - | - | | - | 161.373 | 60 | 208.566 | 62.8 | 0 | |
| Multiple meta-predictors model | | | |  |  |  |  |  |  | |
| SO_2_+  Pop_Growth | 10.277 | 6 | | 0.113 | 139.034 | 54 | 210.289 | 61.2 | -1.6 | |

Table S7 Significant effect modifiers of long-term air pollution indicators on the relationship of HFMD with short-term exposure to meteorological factors and air pollution according to the multivariate meta-regression analysis in the Sichuan Basin.

| Exposure | Modifier | LR test | | | Cochran Q test | | Model fit | Heterogeneity | |
| --- | --- | --- | --- | --- | --- | --- | --- | --- | --- |
|  |  | stats | df | *P* value | Q | df | AIC | I^2^ | ΔI^2^ |
| Relative humidity | SO_2_ | 12.960 | 3 | 0.005 | 99.222 | 45 | 147.973 | 54.6 | -3.8 |
|  | reference | - | - | - | 115.360 | 48 | 154.933 | 58.4 | 0 |
| Wind velocity | SO_2_ | 9.858 | 3 | 0.020 | 91.315 | 45 | 175.588 | 50.7 | -4.2 |
|  | CO | 8.722 | 3 | 0.033 | 92.353 | 45 | 176.724 | 51.3 | -3.6 |
|  | reference | - | - | - | 106.332 | 48 | 179.446 | 54.9 | 0 |

**Text S1. Sensitivity Analysis**

1. The choice of the lag structure of environmental factors on HFMD.

To explore the whole lag structure of environmental factors, we used a series of lag settings for up to 30 days to construct several unconstrained distributed lag models for the 21 cities. Firstly, as shown in Fig. S5, the average estimates of the six environmental factors after a lag of 17 days for the 21 cities gradually decreased to zero or then regularly fluctuated around zero, which may be negligible. Secondly, considering the incubation period (3-5 days) and the approximately two-week infection period for HFMD, 17 days was chosen to reflect the lag structure of environmental factors on HFMD based on the natural history of HFMD infection.

Splines with 3-5 dfs were commonly used to capture the lag distribution when modeling the relationship between environmental factors and infectious diseases. As our main focus is on shaping the exposure-response relationship instead of the lag-response relationship, we chose natural cubic 4-dfs splines, which seem sufficient to express the complexity of the lag distribution according to the figure of the lag structure (Fig. S5). Therefore, we used natural cubic splines for lag effects with 4 dfs in the final model.

Sensitivity analysis was conducted by using max 7-21 lag days (Fig. S6), 3-5 df for lag-response (Fig. S7), and by changing the location of knots for lag-response (Fig. S8) after the model structure was completely determined. The results showed that the patterns of the exposure-response curve were little affected.


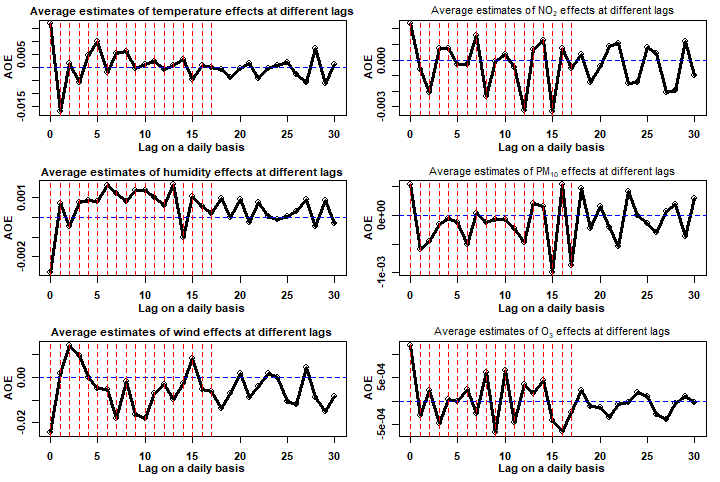


Fig. S5. Average estimates of environmental factors effects at different lags (up to 30 days).


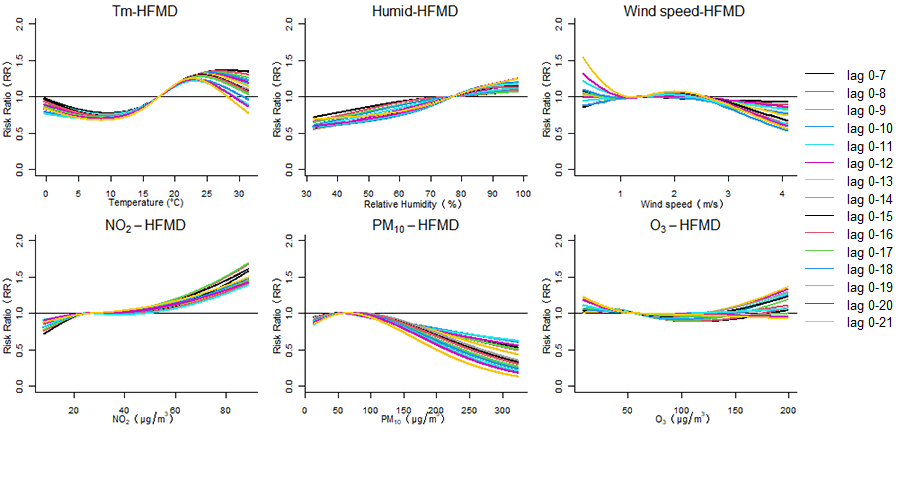


Fig. S6. Overall relationship of HFMD counts with meteorological factors and air pollution at different lag intervals.


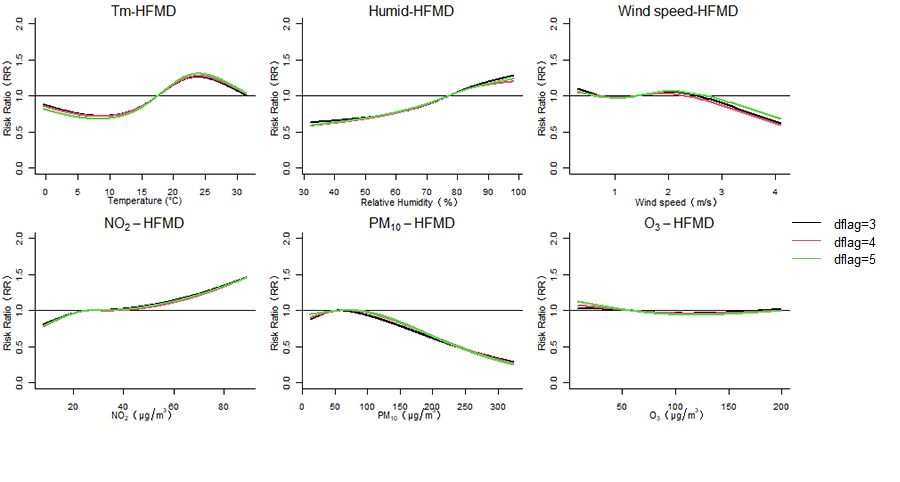


Fig. S7. Overall relationship of HFMD counts with meteorological factors and air pollution at different splines with 3-5 dfs for lag-response.


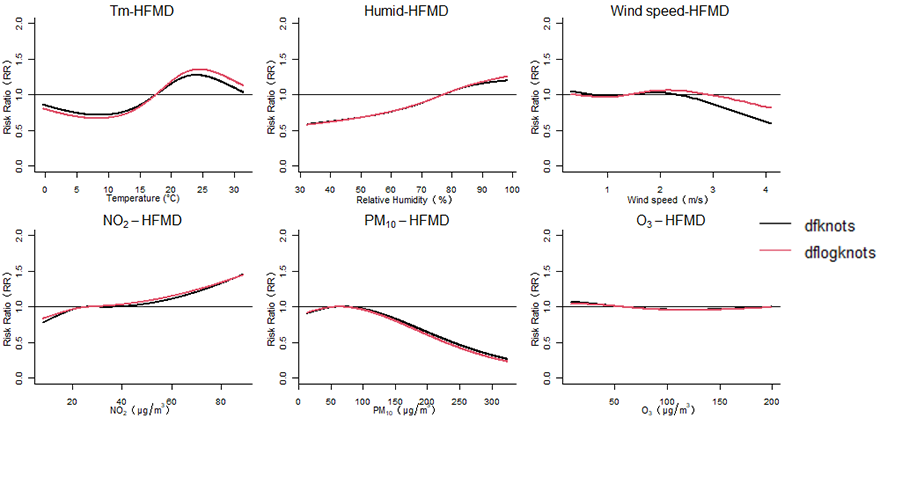


Fig. S8. Overall relationship of HFMD counts with meteorological factors and air pollution at different locations of knots for lag-response.

2. The choice of the parameters that define the environmental factors-HFMD relationship.

We performed a systematic sensitivity analysis to determine the dfs of natural cubic splines for the effects of environmental factors by varying the dfs from 3 to 8. With the increase of dfs, the shapes for the environmental factors-HFMD curves became more flexible (Fig. S9). The QAICs values of the DLNM increases until it peaked at 6dfs (Table S8). Considering the interpretability of existing biological mechanisms and the goodness of model fit, we used natural cubic splines for the effects of environmental factors with the dfs set to 3.

Table S8 The determination of dfs of spline function for exposure-response.

| dfs | Q-AICs |
| --- | --- |
| 3 | 103246.7 |
| 4 | 103324.9 |
| 5 | 103311.1 |
| 6 | 103365.9 |
| 7 | 103137.8 |
| 8 | 103036.7 |


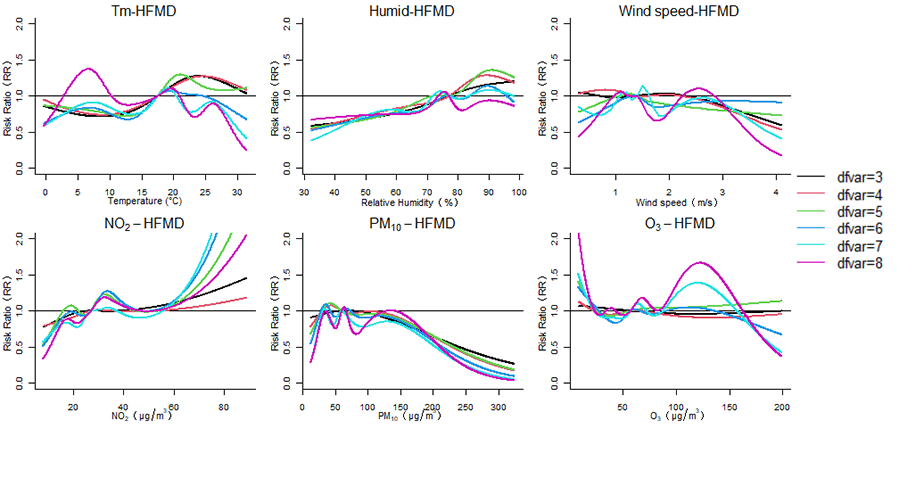


Fig. S9. Overall relationship of HFMD counts with meteorological factors and air pollution at different splines with 3-8 dfs for exposure-response.

Table S9 Significant modification effects of long-term air pollution indicators on the relationship of HFMD with short-term exposure to meteorological factors and air pollution at different splines with 3-8 dfs for exposure-response.

| Significant modification effects | dfvar=3 | dfvar=4 | dfvar=5 | dfvar=6 | dfvar=7 | dfvar=8 |
| --- | --- | --- | --- | --- | --- | --- |
| SO_2_ modify Tm-HFMD | ● |  | ● | ● | ● | ● |
| CO modify Tm-HFMD | ● |  |  | ● | ● | ● |
| PM_10_ modify Tm-HFMD |  | ● |  |  |  |  |
| SO_2_ modify Humid-HFMD | ● | ● | ● |  |  |  |
| CO modify Humid-HFMD |  |  | ● |  |  |  |
| PM_10_ modify Humid-HFMD |  |  | ● | ● |  |  |
| NO_2_ modify Wind-HFMD |  |  |  | ● | ● | ● |
| CO modify Wind-HFMD | ● |  |  |  |  |  |
| PM_10_ modify Wind-HFMD |  |  |  | ● |  |  |
| O_3_ modify NO_2_-HFMD |  |  |  |  | ● |  |
| PM_10_ modify NO_2_-HFMD |  |  |  | ● |  |  |
| O_3_ modify PM_10_-HFMD |  |  | ● |  |  |  |

In addition, we have changed the location of knots for exposure-response (equally spaced values, 30th and 70th percentiles for exposure, 20th and 80th percentiles for exposure, 15th and 85th percentiles for exposure, 10th and 90th percentiles for exposure). The shapes of the exposure-response curves and significant effect modifiers were robust (Fig. S10, Table S11). We found that the best goodness of model fit was achieved at the natural cubic splines model with equally spaced knots (Table S10). Therefore, we placed the knots for the splines of exposure-response at equally spaced values in the final model.

Table S10 The determination of location of knots for exposure-response.

| knots | Q-AICs |
| --- | --- |
| equalknots | 103246.7 |
| 30th-70th | 103248.2 |
| 20th-80th | 103265.7 |
| 15th-85th | 103271.8 |
| 10th-90th | 103278.7 |


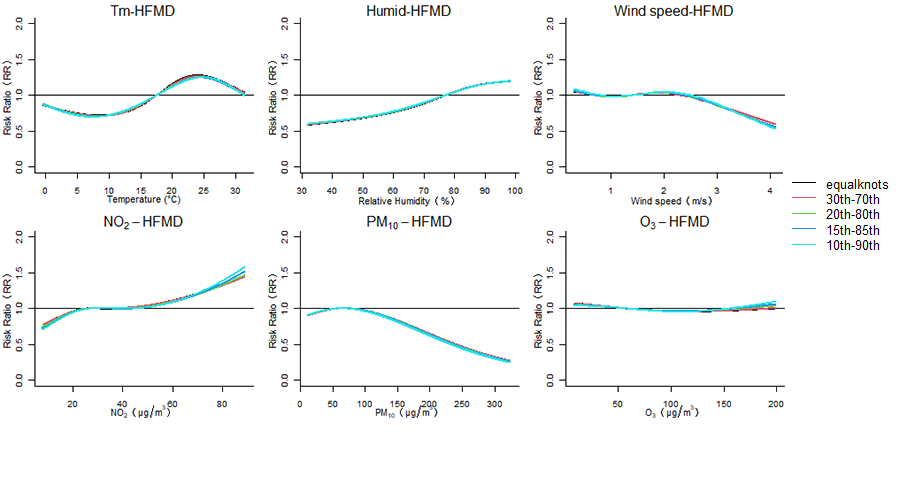


Fig. S10. Overall relationship of HFMD counts with meteorological factors and air pollution at different location of knots for exposure-response.

Table S11 Significant modification effects of long-term air pollution indicators on the relationship of HFMD with short-term exposure to meteorological factors and air pollution at different location of knots for exposure-response.

| knots | SO_2_ modify  Tm-HFMD | CO modify  Tm-HFMD | SO_2_ modify  Humid-HFMD | CO modify  Wind-HFMD |
| --- | --- | --- | --- | --- |
| equalknots | ● | ● | ● | ● |
| 30th-70th | ● | ● | ● | ● |
| 20th-80th |  | ● | ● |  |
| 15th-85th |  | ● | ● |  |
| 10th-90th |  | ● | ● |  |

3. A comparative analysis of expose-response associations at specific lags (lag0 and lag16).

The exposure-lag-response relationships were reduced to the unidimensional exposure-response at lag0 and lag16. Then we applied the multivariate meta-regression to pooled the lag-specific associations. The RR of lag0 was generally higher than that of lag16, while the effects of temperature, PM_10_ and O_3_ on HFMD were still significant in some particular intervals of exposure at lag16 (Fig. S11-12).


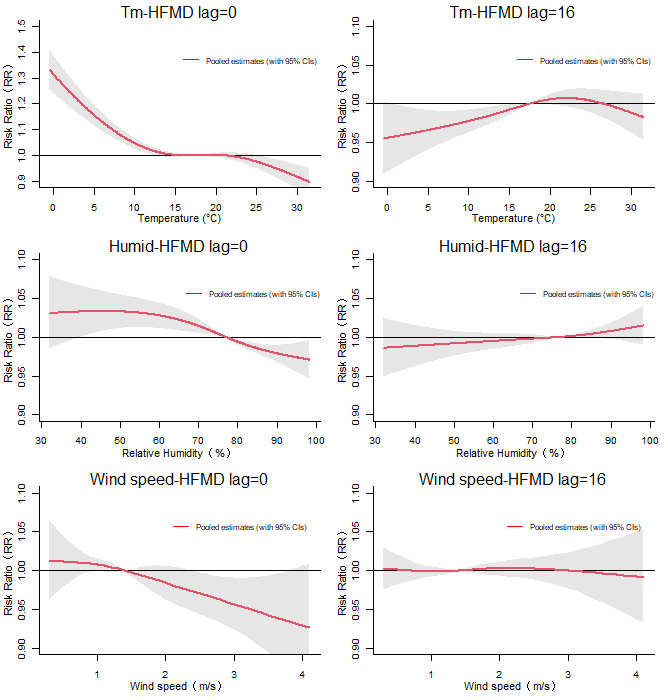


Fig. S11. Pooled relationship of HFMD counts with meteorological factors at lag0 and lag16.


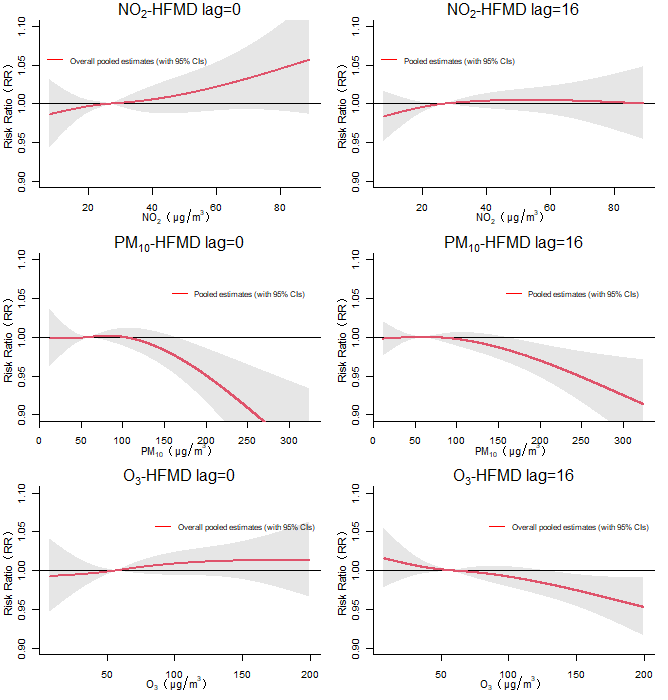


Fig. S12. Pooled relationship of HFMD counts with air pollution at lag0 and lag16.
